# Silver indium diphosphate, AgInP_2_O_7_

**DOI:** 10.1107/S1600536810052451

**Published:** 2010-12-18

**Authors:** Hafid Zouihri, Mohamed Saadi, Boujemaa Jaber, Lehcen El Ammari

**Affiliations:** a Laboratoire de Chimie du Solide Appliquée, Faculté des Sciences, Université Mohammed V-Agdal, Avenue Ibn Batouta, BP 1014, Rabat, Morocco; b Centre National pour la Recherche Scientifique et Technique, Division UATRS, Angle Allal AlFassi et Avenue des FAR, Hay Ryad, BP 8027, Rabat, Morocco

## Abstract

Polycrystalline material of the title compound, AgInP_2_O_7_, was synthesized by traditional high-temperature solid-state methods and single crystals were grown from the melt of a mixture of AgInP_2_O_7_ and B_2_O_3_ as flux in a platinium crucible. The structure consists of InO_6_ octa­hedra, which are corner-shared to PO_4_ tetra­hedra into a three-dimensional network with hexa­gonal channels running parallel to the *c* axis. The silver cation, located in the channel, is bonded to seven O atoms of the [InP_2_O_7_] framework with Ag–O distances ranging from 2.370 (2) to 3.015 (2) Å. The P_2_O_7_ diphosphate anion is characterized by a P—O—P angle of 137.27 (9) and a nearly eclipsed conformation. AgInP_2_O_7_ is isotypic with the *M* ^I^FeP_2_O_7_ (*M* ^I^ = Na, K, Rb, Cs and Ag) diphosphate family.

## Related literature

For properties of *M* ^I^FeP_2_O_7_ (*M* ^I^ = Na, K, Rb, Cs and Ag) diphosphates, see: Terebilenko *et al.* (2010 ▶); Hizhnyi *et al.* (2008 ▶); Whangbo *et al.* (2004 ▶); Vitins *et al.* (2000 ▶). For isotypic structures, see: Belkouch *et al.* (1995 ▶); Gabelica-Robert *et al.* (1982 ▶); Moya-Pizarro *et al.* (1984 ▶); Mercader *et al.* (1990 ▶).

## Experimental

### 

#### Crystal data

AgInP_2_O_7_
*M* *_r_* = 396.63
Monoclinic, 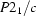

*a* = 7.4867 (3) Å
*b* = 8.2620 (3) Å
*c* = 9.8383 (5) Å
β = 112.038 (2)°
*V* = 564.09 (4) Å^3^
*Z* = 4
Mo *K*α radiation
μ = 8.11 mm^−1^
*T* = 296 K
0.08 × 0.06 × 0.05 mm

#### Data collection

Bruker X8 APEXII CCD area-detector diffractometer
Absorption correction: multi-scan (*SADABS*; Sheldrick, 1999 ▶) *T*_min_ = 0.563, *T*_max_ = 0.667
21692 measured reflections
3730 independent reflections
3245 reflections with *I* > 2σ(*I*)
*R*_int_ = 0.035

#### Refinement

*R*[*F* ^2^ > 2σ(*F* ^2^)] = 0.021
*wR*(*F* ^2^) = 0.048
*S* = 1.03
3730 reflections
101 parameters
Δρ_max_ = 1.62 e Å^−3^
Δρ_min_ = −2.04 e Å^−3^

### 

Data collection: *APEX2* (Bruker, 2005 ▶); cell refinement: *SAINT* (Bruker, 2005 ▶); data reduction: *SAINT*; program(s) used to solve structure: *SHELXS97* (Sheldrick, 2008 ▶); program(s) used to refine structure: *SHELXL97* (Sheldrick, 2008 ▶); molecular graphics: *ORTEP-3 for Windows* (Farrugia, 1997 ▶); software used to prepare material for publication: *WinGX* (Farrugia, 1999 ▶).

## Supplementary Material

Crystal structure: contains datablocks I, global. DOI: 10.1107/S1600536810052451/br2154sup1.cif

Structure factors: contains datablocks I. DOI: 10.1107/S1600536810052451/br2154Isup2.hkl

Additional supplementary materials:  crystallographic information; 3D view; checkCIF report

## Figures and Tables

**Fig. 1.**
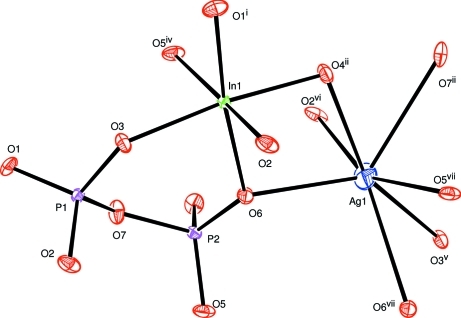
Partial plot of AgInP2O7 crystal structure shawing plyhedra linkage. Displacement ellipsoids are drawn at the 50% probability level. Symmetry codes: (i) -x + 1, y - 1/2, -z + 1/2; (ii) -x, y - 1/2, -z + 1/2; (iii) -x + 1, -y + 1, -z + 1; (iv) x, -y + 3/2, z - 1/2; (v) x - 1, y, z; (vi) x - 1, -y + 3/2, z - 1/2; (vii) -x, -y + 1, -z + 1.

**Fig. 2.**
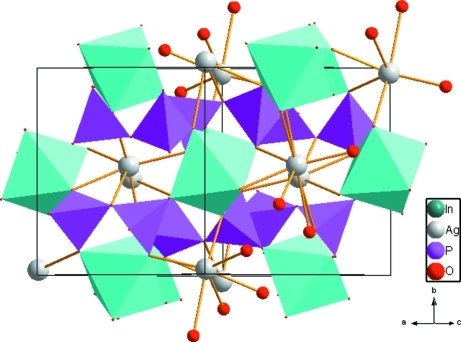
Perspective view along [101] of the AgInP2O7 framework structure shawing tunnel where silver cations are located.

## supplementary crystallographic information

### Comment

The diphosphates A^I^*M*^III^P_2_O_7_ (A^I^ = Li, Na, K, Rb, Cs and Ag; *M*^III^ = Al, Ga, Cr, Fe, In, Y) are extensively studied for their electrical and optical properties and for its perspective application as magnetic materials (Terebilenko *et al.* (2010); Hizhnyi *et al.* (2008); Whangbo *et al.* (2004); Vitins *et al.* (2000)). The crystal structures of most of these compounds are known except a few cases in which the crystal growth is difficult. In this context, the present paper reports on the determination of AgInP_2_O_7_ crystal structure from X-ray diffraction single-crystal data.

The structure of this phosphate is characterized by a three-dimensional network built up from indum octahedra linked to diphosphate groups by a corner-sharing. Each InO_6_ octahedra is surrounded by six PO_4_ tetrahedra belonging to five different P_2_O_7_ groups (see Fig.1 and Fig.2). As a result of these blocks, assemblage three-dimensional-framework is formed with hexagonal channels, where silver cations reside. Although, the coordination sphere of Ag^+^ cations is composed of seven O^2-^ anions in an irregular geometry, located at Ag–O distances between 2.370 (2) and 3.015 (2) Å (see Fig.2). Furthermore, the diphosphate group contains two distorted PO_4_ tetrahedra sharing one corner and display a nearly eclipsed conformation. The P–O bond-lengths range between 1.492 (2) Å for terminal P1–O1 and 1.606 (2) Å for the bridging P2–O7 bond. Therefore, a P1–O7–P2 angle of 137.27 (9) ° is wider than 133.6 (3)° and 132.9 (3) ° reported for both AgFeP_2_O_7_ and NaFeP_2_O_7_ respectively (Belkouch *et al.* (1995); Gabelica-Robert *et al.* (1982); Moya-Pizarro *et al.* (1984); Mercader *et al.* (1990)).

Silver indium diphosphate (pyrophosphate) is isostructural to A^I^FeP_2_O_7_ (A^I^ = Na, K, Rb, Cs and Ag) diphosphates family and is categorized as a dichromate type.

### Experimental

AgInP_2_O_7_ in the form of single crystals was prepared by stoichiometric reaction of AgNO_3_, (NH_4_)_2_HPO_4_ and In_2_O_3_ in B_2_O_3_ flux. The mixture was heated at 773 K under ambiante atmosphere for 6 h and 973 K for 2 h with intermediate grindings to ensure complete reaction. Subsequent melting at 1323 K followed by slow cooling to room temperature at a rate of 12°K h^-1^ resulted in colourless crystals of the title compound.

### Refinement

The highest and deepest hole residual peak in the final difference Fourier map are located at 0.49 Å and 0.58 Å, respectively from Ag1 atom. The not significants bonds and angles were removed from the CIF file.

### Crystal data

**Table:**

AgInP_2_O_7_	*F*(000) = 728
*M**_r_* = 396.63	*D*_x_ = 4.670 Mg m^−^^3^
Monoclinic, *P*2_1_/*c*	Mo *K*α radiation, λ = 0.71073 Å
Hall symbol: -P 2ybc	Cell parameters from 317 reflections
*a* = 7.4867 (3) Å	θ = 2.5–30.2°
*b* = 8.2620 (3) Å	µ = 8.11 mm^−^^1^
*c* = 9.8383 (5) Å	*T* = 296 K
β = 112.038 (2)°	Block, colourless
*V* = 564.09 (4) Å^3^	0.08 × 0.06 × 0.05 mm
*Z* = 4	

### Data collection

**Table:**

Bruker X8 APEXII CCD area-detector diffractometer	3730 independent reflections
Radiation source: fine-focus sealed tube	3245 reflections with *I* > 2σ(*I*)
graphite	*R*_int_ = 0.035
ω and φ scans	θ_max_ = 41.0°, θ_min_ = 2.9°
Absorption correction: multi-scan (*SADABS*; Sheldrick, 1999)	*h* = −13→13
*T*_min_ = 0.563, *T*_max_ = 0.667	*k* = −15→15
21692 measured reflections	*l* = −18→18

### Refinement

**Table:**

Refinement on *F*^2^	Primary atom site location: structure-invariant direct methods
Least-squares matrix: full	Secondary atom site location: difference Fourier map
*R*[*F*^2^ > 2σ(*F*^2^)] = 0.021	*w* = 1/[σ^2^(*F*_o_^2^) + (0.017*P*)^2^ + 0.9979*P*] where *P* = (*F*_o_^2^ + 2*F*_c_^2^)/3
*wR*(*F*^2^) = 0.048	(Δ/σ)_max_ = 0.001
*S* = 1.03	Δρ_max_ = 1.62 e Å^−^^3^
3730 reflections	Δρ_min_ = −2.04 e Å^−^^3^
101 parameters	Extinction correction: *SHELXL97* (Sheldrick, 2008), Fc^*^=kFc[1+0.001xFc^2^λ^3^/sin(2θ)]^-1/4^
0 restraints	Extinction coefficient: 0.0171 (4)

### Special details

**Table:**

Geometry. All s.u.'s (except the s.u. in the dihedral angle between two l.s. planes) are estimated using the full covariance matrix. The cell s.u.'s are taken into account individually in the estimation of s.u.'s in distances, angles and torsion angles; correlations between s.u.'s in cell parameters are only used when they are defined by crystal symmetry. An approximate (isotropic) treatment of cell s.u.'s is used for estimating s.u.'s involving l.s. planes.
Refinement. Refinement of *F*^2^ against ALL reflections. The weighted *R*-factor *wR* and goodness of fit *S* are based on *F*^2^, conventional *R*-factors *R* are based on *F*, with *F* set to zero for negative *F*^2^. The threshold expression of *F*^2^ > σ(*F*^2^) is used only for calculating *R*-factors(gt) *etc*. and is not relevant to the choice of reflections for refinement. *R*-factors based on *F*^2^ are statistically about twice as large as those based on *F*, and *R*- factors based on ALL data will be even larger.

### Fractional atomic coordinates and isotropic or equivalent isotropic displacement parameters (Å^2^)

**Table:**

	*x*	*y*	*z*	*U*_iso_*/*U*_eq_	
In1	0.242354 (15)	0.495357 (12)	0.247622 (11)	0.00618 (3)	
Ag1	−0.20911 (3)	0.52697 (2)	0.30478 (2)	0.02442 (4)	
P1	0.57689 (6)	0.74758 (5)	0.46083 (4)	0.00600 (6)	
P2	0.17589 (6)	0.78735 (5)	0.45174 (4)	0.00656 (6)	
O1	0.6810 (2)	0.86792 (17)	0.40464 (15)	0.0141 (2)	
O2	0.6836 (2)	0.71622 (16)	0.62241 (14)	0.0136 (2)	
O3	0.52473 (17)	0.59259 (15)	0.36935 (14)	0.01027 (19)	
O4	0.04427 (18)	0.91166 (17)	0.35059 (15)	0.0123 (2)	
O5	0.1917 (2)	0.79561 (16)	0.60976 (14)	0.0126 (2)	
O6	0.13231 (18)	0.61348 (15)	0.39564 (14)	0.01054 (19)	
O7	0.37868 (18)	0.83601 (16)	0.44239 (16)	0.0128 (2)	

### Atomic displacement parameters (Å^2^)

**Table:**

	*U*^11^	*U*^22^	*U*^33^	*U*^12^	*U*^13^	*U*^23^
In1	0.00656 (4)	0.00614 (4)	0.00593 (4)	−0.00024 (3)	0.00244 (3)	−0.00045 (3)
Ag1	0.01913 (7)	0.02720 (8)	0.03341 (9)	−0.00235 (6)	0.01725 (7)	−0.01071 (7)
P1	0.00574 (14)	0.00643 (14)	0.00582 (14)	−0.00025 (11)	0.00217 (11)	0.00044 (11)
P2	0.00588 (14)	0.00706 (14)	0.00644 (14)	0.00073 (11)	0.00195 (11)	−0.00090 (12)
O1	0.0185 (6)	0.0141 (5)	0.0133 (5)	−0.0053 (4)	0.0104 (5)	0.0012 (4)
O2	0.0195 (6)	0.0098 (5)	0.0070 (4)	−0.0006 (4)	−0.0001 (4)	0.0018 (4)
O3	0.0072 (4)	0.0099 (5)	0.0130 (5)	−0.0012 (3)	0.0030 (4)	−0.0040 (4)
O4	0.0084 (5)	0.0135 (5)	0.0135 (5)	0.0034 (4)	0.0023 (4)	0.0041 (4)
O5	0.0211 (6)	0.0099 (5)	0.0072 (4)	−0.0009 (4)	0.0058 (4)	−0.0023 (4)
O6	0.0116 (5)	0.0097 (5)	0.0123 (5)	−0.0019 (4)	0.0067 (4)	−0.0039 (4)
O7	0.0078 (5)	0.0100 (5)	0.0217 (6)	0.0010 (4)	0.0069 (4)	−0.0020 (4)

### Geometric parameters (Å, °)

**Table:**

In1—O1^i^	2.0799 (13)	Ag1—O5^vii^	2.7829 (14)
In1—O4^ii^	2.1120 (12)	Ag1—O6^vii^	3.0153 (14)
In1—O2^iii^	2.1133 (13)	P1—O1	1.4919 (13)
In1—O5^iv^	2.1401 (13)	P1—O2	1.5097 (13)
In1—O3	2.1562 (12)	P1—O3	1.5292 (13)
In1—O6	2.1569 (12)	P1—O7	1.6021 (13)
Ag1—O3^v^	2.3703 (12)	P2—O4	1.5101 (13)
Ag1—O6	2.4757 (13)	P2—O5	1.5158 (13)
Ag1—O4^ii^	2.4865 (14)	P2—O6	1.5295 (13)
Ag1—O2^vi^	2.6991 (14)	P2—O7	1.6062 (13)
Ag1—O7^ii^	2.7744 (15)		
			
O1^i^—In1—O4^ii^	90.70 (6)	O3^v^—Ag1—O5^vii^	94.99 (4)
O1^i^—In1—O2^iii^	86.35 (6)	O6—Ag1—O5^vii^	104.03 (4)
O4^ii^—In1—O2^iii^	89.82 (6)	O4^ii^—Ag1—O5^vii^	80.85 (4)
O1^i^—In1—O5^iv^	89.04 (5)	O2^vi^—Ag1—O5^vii^	157.25 (4)
O4^ii^—In1—O5^iv^	93.79 (5)	O7^ii^—Ag1—O5^vii^	71.00 (4)
O2^iii^—In1—O5^iv^	174.18 (6)	O3^v^—Ag1—O6^vii^	72.37 (4)
O1^i^—In1—O3	96.36 (5)	O6—Ag1—O6^vii^	88.04 (4)
O4^ii^—In1—O3	172.86 (5)	O4^ii^—Ag1—O6^vii^	119.79 (4)
O2^iii^—In1—O3	89.55 (5)	O2^vi^—Ag1—O6^vii^	148.46 (4)
O5^iv^—In1—O3	87.43 (5)	O7^ii^—Ag1—O6^vii^	119.64 (4)
O1^i^—In1—O6	173.56 (5)	O5^vii^—Ag1—O6^vii^	50.65 (4)
O4^ii^—In1—O6	82.94 (5)	O1—P1—O2	111.17 (8)
O2^iii^—In1—O6	92.62 (5)	O1—P1—O3	113.17 (8)
O5^iv^—In1—O6	92.35 (5)	O2—P1—O3	113.16 (8)
O3—In1—O6	89.98 (5)	O1—P1—O7	104.17 (8)
O3^v^—Ag1—O6	134.04 (4)	O2—P1—O7	107.40 (8)
O3^v^—Ag1—O4^ii^	155.99 (4)	O3—P1—O7	107.10 (7)
O6—Ag1—O4^ii^	69.47 (4)	O4—P2—O5	115.20 (8)
O3^v^—Ag1—O2^vi^	85.86 (5)	O4—P2—O6	113.76 (8)
O6—Ag1—O2^vi^	91.29 (4)	O5—P2—O6	109.65 (8)
O4^ii^—Ag1—O2^vi^	89.15 (5)	O4—P2—O7	100.90 (8)
O3^v^—Ag1—O7^ii^	102.17 (4)	O5—P2—O7	109.63 (8)
O6—Ag1—O7^ii^	123.47 (4)	O6—P2—O7	107.01 (7)
O4^ii^—Ag1—O7^ii^	54.04 (4)	P1—O7—P2	137.27 (9)
O2^vi^—Ag1—O7^ii^	86.57 (4)		

Symmetry codes: (i) −*x*+1, *y*−1/2, −*z*+1/2; (ii) −*x*, *y*−1/2, −*z*+1/2; (iii) −*x*+1, −*y*+1, −*z*+1; (iv) *x*, −*y*+3/2, *z*−1/2; (v) *x*−1, *y*, *z*; (vi) *x*−1, −*y*+3/2, *z*−1/2; (vii) −*x*, −*y*+1, −*z*+1.
